# Acetamiprid Accumulates in Different Amounts in Murine Brain Regions

**DOI:** 10.3390/ijerph13100937

**Published:** 2016-09-22

**Authors:** Hayato Terayama, Hitoshi Endo, Hideo Tsukamoto, Koichi Matsumoto, Mai Umezu, Teruhisa Kanazawa, Masatoshi Ito, Tadayuki Sato, Munekazu Naito, Satoshi Kawakami, Yasuhiro Fujino, Masayuki Tatemichi, Kou Sakabe

**Affiliations:** 1 Department of Anatomy, Division of Basic Medicine, Tokai University School of Medicine, Kanagawa 259-1193, Japan; smiles-and-tears.3ks@hotmail.co.jp (K.M.); 3bhn1127@mail.tokai-u.jp (M.U.); book0822@gmail.com (T.K.); sakabek@tokai-u.jp (K.S.); 2 Department of Community Health, Tokai University School of Medicine, Kanagawa 259-1193, Japan; h-endo@tokai-u.jp (H.E.); tatemichi@tokai-u.jp (M.T.); 3 Support Center for Medical Research and Education, Tokai University School of Medicine, Kanagawa 259-1193, Japan; HTsukamoto@tsc.u-tokai.ac.jp (H.T.); ito@tokai-u.jp (M.I.); tadayuki@is.icc.u-tokai.ac.jp (T.S.); 4 Department of Anatomy, Aichi Medical University, Aichi 480-1195, Japan; munekazunaito@gmail.com; 5 Division of Oral and Craniofacial Anatomy, Graduate School of Dentistry, Tohoku University, Sendai 980-8575, Japan; ksato21@dent.tohoku.ac.jp; 6 Department of Human Development, Tokai University School of Humanities and Culture, Kanagawa 259-1292, Japan; yfujino@keyaki.cc.u-tokai.ac.jp

**Keywords:** acetamiprid, mouse, brain, concentration, neonicotinoid

## Abstract

Neonicotinoids such as acetamiprid (ACE) belong to a new and widely used single class of pesticides. Neonicotinoids mimic the chemical structure of nicotine and share agonist activity with the nicotine acetylcholine receptor (nAchR). Neonicotinoids are widely considered to be safe in humans; however, they have recently been implicated in a number of human health disorders. A wide range of musculoskeletal and neuromuscular disorders associated with high doses of neonicotinoids administered to animals have also been reported. Consequently, we used a mouse model to investigate the response of the central nervous system to ACE treatment. Our results show that exposure to ACE-containing water for three or seven days (decuple and centuple of no observable adverse effect level (NOAEL)/day) caused a decrease in body weight in 10-week old A/JJmsSlc (A/J) mice. However, the treatments did not affect brain histology or expression of CD34. ACE concentrations were significantly higher in the midbrain of ACE-treated mice than that of the normal and vehicle groups. Expression levels of α7, α4, and β2 nAChRs were found to be low in the olfactory bulb and midbrain of normal mice. Furthermore, in the experimental group (centuple ACE-containing water for seven days), β2 nAChR expression decreased in many brain regions. Information regarding the amount of accumulated ACE and expression levels of the acetylcholine receptor in each region of the brain is important for understanding any clinical symptoms that may be associated with ACE exposure.

## 1. Introduction

The first major neonicotinoids used as pesticides were chloropyridylmethyl compounds (imidacloprid, nitenpyram, thiacloprid, and acetamiprid). This was followed soon thereafter by the chlorothiazolylmethyl insecticides (thiamethoxam and clothianidin) and a tetrahydrofuranylmethyl analog (dinotefuran) [1,2]. The insecticidal activity of neonicotinoids is primarily attributed to their action on nicotinic acetylcholine receptors (nAChRs) [3,4]. The acute toxicity of neonicotinoids is attributed primarily to their action as nicotinic agonists, acting on insect and mammal nAChRs [5].

Mammalian nAChRs exist as heteromeric complexes, comprising α (α2–α6) and β (β2–β4) subunits, or as homomeric complexes, comprising α (α7–α9) subunits [6]. However, a few subtypes of nAChRs predominate, notably α4β2 and α7, which are widespread in the vertebrate central nervous system (CNS) [7,8]. Neonicotinoid toxicity in mammalians correlates well with agonist action and binding affinity at the α4β2 and α7 nAChRs, which are the primary targets for neonicotinoids in the brain [4,9,10].

Acetamiprid (ACE) is a broad-spectrum insecticide that belongs to the neonicotinoid class. Upon oral ingestion, ACE is absorbed at levels of almost 100% in the intestinal tract [11]. After being metabolized by liver cytochrome P450 (CYP) enzymes, ACE is excreted in the feces and urine. Shi et al. [12] reported that concentrations of clothianidin and thiacloprid increase in the brain and liver when CYP enzymes are inhibited. Ford et al. [1,2] also reported that ACE and its metabolic products were observed in brain, liver, plasma, and urine from ACE-treated rats. Thus, it would appear that ACE can pass through the blood-brain barrier. However, to our knowledge, there are no reports that have investigated in vivo accumulation of neonicotinoid insecticides in each of the different regions of the mammalian brain.

From a clinical perspective, there were 1142 neonicotinoid exposures reported to poison centers in Texas, USA from 2000 to 2012 [13]; most were associated with products containing imidacloprid (77%) or dinotefuran (17%). Exposure to ACE was associated with only 0.5% of the reported incidents. The most commonly reported adverse clinical effects of neonicotinoid exposure were ocular irritation, dermal irritation, nausea, vomiting, oral irritation, erythema, and red eye.

On the other hand, the food residue limit for ACE in Japan is set higher than that of Europe and the United States [14]. Cases of acute ACE poisoning have been reported in Japan [15,16,17,18]. The most commonly reported adverse clinical effects include cardiovascular symptoms, central nervous symptoms, respiratory symptoms, low body temperature, muscle weakness, miosis, and dry mouth [15,16,17,18], suggesting a wide variety of possible clinical symptoms associated with ACE poisoning. These clinical symptoms are related directly and/or indirectly to effects on the central nervous system. In the present study, we investigated the accumulation of ACE in different brain regions of ACE-exposed mice. We further evaluated expression levels of nAChRs, the major binding target of ACE, in each of the different brain regions of vehicle mice.

## 2. Materials and Methods

### 2.1. Ethics Statement

The animal study was approved by the institutional review board of Tokai University School of Medicine (141003) (Kanagawa, Japan).

### 2.2. Animals

Male A/JJmsSlc (A/J) mice (8 weeks old) were purchased from SLC (Shizuoka, Japan) and kept at the Department of Laboratory Animal Science, the Support Center for Medical Research and Education, Tokai University for 1 week. They were kept at 22–24 °C and 50%–60% relative humidity on a 12 h light, 12 h dark cycle. The animals were fed a standard commercial diet (Clea, Tokyo, Japan) and allowed to drink water during the entire experimental period.

### 2.3. Chemicals

Mospilan SP containing 18% ACE was purchased from Nippon Soda Co., Ltd. (Tokyo, Japan). The different doses of Mospilan SP used in this study were separately dissolved in water. All mice were 8 weeks of age at the start of the experiments, and the diet and drinking water were freely available. The no observed adverse effect level (NOAEL) of ACE in rat is known to be 7.1 mg/kg/day; therefore, in these studies, we used doses of acetamiprid E1 at a ten-fold volume of NOAEL (71 μg/g/day) and acetamiprid E2 at a hundred-fold volume of NOAEL (710 μg/g/day) [19].

### 2.4. Experimental Design

Mice were randomly allocated into normal, vehicle, acetamiprid E1, and acetamiprid E2 groups. The normal group was fed standard water for 3 and 7 days. The vehicle group was fed standard water containing surfactant agent (dimethyl sulfoxide) for 3 and 7 days. ACE contained in Mospilan SP is dissolved in various surfactants. Because dimethyl sulfoxide is the most concentrated surfactant found in Mospilan SP, we used it as the vehicle control in these studies. The acetamiprid E1 group was fed water containing ACE for 3 and 7 days, as was the acetamiprid E2 group. Food and water were freely available. On average, the mice ate 4.46 ± 0.43, 4.34 ± 0.36, 4.02 ± 0.27, and 3.93 ± 0.32 mL/mouse/day (mean ± standard deviations (SD)) of water in the normal, vehicle, acetamiprid E1, and acetamiprid E2 groups, respectively. The mice in all four groups ate approximately 5 g of the diet per day. At the end of the study, the mice were fully anesthetized with isoflurane (3%–4%), and their body weights were recorded. Serum samples were taken from the hearts of the mice. Mice were killed by cervical dislocation. The brains were removed and the cerebral cortex and medulla, diencephalon, midbrain, medulla oblongata, cerebellum, hippocampus, corpus striatum, and the olfactory bulb were grossly isolated. Each region of the brain was evaluated histologically using a hematoxylin and eosin (H & E) stain.

### 2.5. Light Microscopy

The cerebral cortex and medulla, diencephalon, midbrain, medulla oblongata, cerebellum, hippocampus, corpus striatum, and the olfactory bulb were removed from mice in the normal (*n* = 5), vehicle (*n* = 5), acetamiprid E1 (*n* = 5), and acetamiprid E2 (*n* = 5) groups at 3 and 7 days. They were fixed in Bouin’s solution for 3 days, washed, dehydrated in an ethanol series, and embedded in plastic (Technovit 7100; Kuizer, Wehrheim, Germany). Each brain region was sectioned at 5 μm with a microtome (HN360; Microm, Waldorf, Germany) and then stained with Gill’s hematoxylin V and eosin Y for observation by light microscopy.

### 2.6. Real-Time RT-PCR

Total RNA was isolated from each brain region of the normal, vehicle, and ACE-treated E2 (3 and 7 days) groups (*n* = 5 for each treatment group) using the TRIzol RNA extraction kit (Invitrogen, Carlsbad, CA, USA) and reverse-transcribed into cDNA using the High-Capacity cDNA Archive Kit (Applied Biosystems, Foster City, CA, USA) according to the manufacturer’s instructions. Quantification of cDNA was performed using the SYBR Premix Ex Taq II (Takara, Otsu, Japan) and StepOnePlus Real-Time PCR System (Applied Biosystems). Primers were used to detect the α4, β2, and α7 nAChRs mRNA (Table S1). β-actin was used as a housekeeping gene to normalize mRNA expression. The relative expression of real-time PCR products was determined using the ΔΔCt method to compare target gene and β-actin mRNA expression.

### 2.7. Western Blot Analysis

The different brain regions from the normal, vehicle, and ACE-treated E2 (3 and 7 days) groups (*n* = 5 from each group) were evaluated for the presence of vascular endothelial cells by Western blot analysis. The concentration of protein was measured with a BCA protein assay kit (Thermo Scientific, Rockford, IL, USA). Samples were mixed with SDS sample buffer (0.25 M Tris-HCl, pH 6.8, 8% SDS, 20% glycerol, 5% beta-mercaptoethanol) and an equal amount of protein per lane was run on a 10% SDS-PAGE gel and transferred onto a polyvinylidene difluoride (PVDF) membrane (Merck Millipore, Berlin, Germany). Blots were incubated with anti-CD34 antibody (Santa Cruz Biotechnology, Dallas, TX, USA) at a dilution of 1:1000 and 4 °C overnight, followed by incubation with horseradish peroxidase-conjugated goat anti-rabbit IgG secondary antibody (Cell Signaling Technology, Danvers, MA, USA) at a dilution of 1:2000 for 1 h at room temperature. The proteins were visualized by chemiluminescence using an ECL Prime Western blotting detection kit (GE Healthcare, Little Chalfont, UK), according to the manufacturer’s instructions. To confirm equal loading of the samples, anti-β-actin antibody (Sigma Aldrich, St. Louis, MO, USA) was used as an internal control. Densitometry analysis was performed with CS Analyzer 3.0 software (ATTO, Tokyo, Japan)—area × optical density and normalized to that of β-actin. The expression levels of target proteins in the treatment groups are reported as fold change over expression levels of target proteins in the control group.

### 2.8. Liquid Chromatography Detection of ACE

For extraction of ACE from tissue, the cerebral cortex and medulla, diencephalon, midbrain, medulla oblongata, cerebellum, hippocampus, corpus striatum, and the olfactory bulb from mice in the normal (*n* = 5), vehicle (*n* = 5), and ACE E2 (*n* = 5) groups (3 and 7 days) were each placed in 80% acetone. A stock solution of standard ACE (160430-64-8, Sigma-Aldrich, St. Louis, MO, USA) was also prepared in 80% acetone. Tissue homogenates were prepared with a shake master (Biomedical Science, Tokyo, Japan) followed by mixing with a vortex. The acetone extract was centrifuged at 2000 *g* for 15 min and then dried using a Savant SPV11 IV centrifugal evaporator (Thermo Fisher Scientific, Waltham, MA, USA). HPLC was conducted using a Shimadzu Prominence system (Shimadzu, Kyoto, Japan) equipped with a quaternary pump, an autosampler, and a column oven. Detection of ACE was based on UV (244 nm). The column was a Capcell Pak AVR column (5 μm, 4.6 i.d. × 250 mm; Shiseido, Tokyo, Japan). The mobile phase was methanol/water (35/65, v/v). The flow rate was 1.0 mL/min, the column oven temperature was 40 °C, and the injection volume was 20 μL. The values were calculated from a calibration curve, and corrected by the tissue weight of each brain region. The limit of detection was 0.1 nmols.

### 2.9. Statistical Analysis

Data are expressed as mean ± standard error (SE). ANOVA and the Tukey post-hoc test were used for statistical analysis. Differences were considered to be statistically significant at *p* < 0.05. Comparisons between two groups were performed using the student’s *t*-test.

## 3. Results

**Figure 1:**
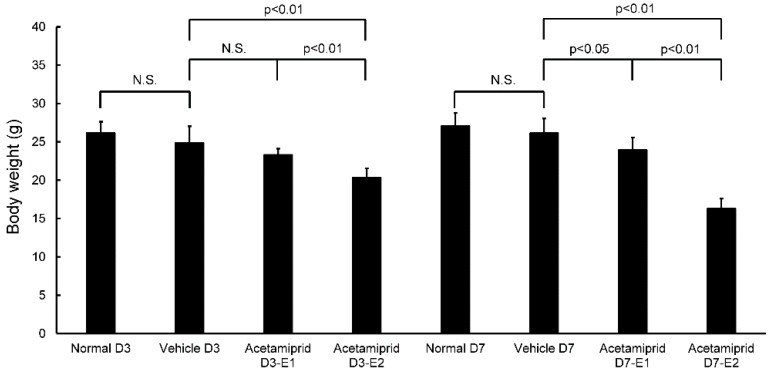
Body weight in normal, vehicle, and acetamiprid-treated mice at days 3 and 7. The normal group was fed standard water. The vehicle group was fed standard water containing surfactant agent (dimethyl sulfoxide without acetamiprid). The dose of acetamiprid E1 was ten-fold that of the No Observed Adverse Effect Level (NOAEL); the dose for acetamiprid E2 was hundred-fold that of the NOAEL. The acetamiprid E1 and acetamiprid E2 groups were fed water containing ACE. At days 3 and 7, the vehicle and normal group were not significantly different from each other. The means ± standard error (SE) are plotted. *p* > 0.05 was considered not significant (N.S.).

Body weights of the ACE E2 mice on day 3 (D3-E2) were significantly lower than the normal and vehicle mice; there were no differences between the ACE E1 mice and the normal and vehicle groups (Figure 1). At day 7, the body weights of both ACE E1 and E2 groups were significantly lower than the normal and vehicle mice (Figure 1). Some mice had diarrhea for the first 3–5 days after initiating treatment. At days 3 and 7, body weights of the normal and vehicle mice were not significantly different from each other. Therefore, for morphological and biochemical analysis, we used the day 7 samples for comparisons between the vehicle and normal mice (non-treated group) and the ACE-treated groups. We observed that the coat of one mouse in the ACE-treated group was in poor condition.

**Figure 2:**
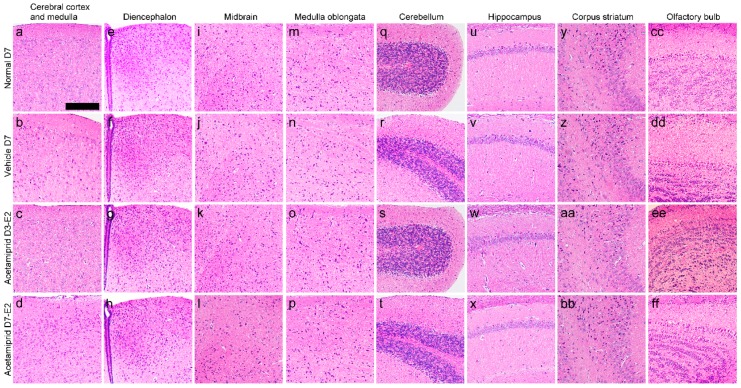
Histological views of various brain tissues of normal, vehicle, and acetamiprid-treated mice. Cerebral cortex and medulla (**a**–**d**); diencephalon (**e**–**h**); midbrain (**i**–**l**); medulla oblongata (**m**–**p**); cerebellum (**q**–**t**); hippocampus (**u**–**x**); corpus striatum (**y**–**bb**); and the olfactory bulb (**cc**–**ff**) of. normal, vehicle, and acetamiprid-treated mice showing intact histological sections. Scale bar = 200 μm.

The histology of the cerebral cortex and medulla (Figure 2a–d), diencephalon (Figure 2e–h), midbrain (Figure 2i–l), medulla oblongata (Figure 2m–p), cerebellum (Figure 2q–t), hippocampus (Figure 2u–x), corpus striatum (Figure 2y–bb), and the olfactory bulb showed no morphological changes in the ACE-treated groups when compared with vehicle and normal mice (the non-treated group).

ACE was not detected in the cerebral cortex and medulla, diencephalon, hippocampus, or corpus striatum of normal mice; it was however detected in the midbrain, medulla oblongata, cerebellum, and the olfactory bulb (Table 1). ACE was not detected in the cerebral cortex and medulla or the corpus striatum of the vehicle mice; it was however detected in the diencephalon, midbrain, medulla oblongata, cerebellum, hippocampus, and the olfactory bulb (Table 1). In the ACE-treated groups (ACE E2 at days 3 and 7), the ACE concentration in each brain region tended to be higher than in the corresponding regions of the normal and vehicle mice (Table 1). In particular, the ACE concentration was significantly higher in the ACE E2 midbrain when compared with that of the normal and vehicle mice (Table 1). ACE accumulation was not significantly different in the various brain regions among the ACE E2 group on day 7 (D7-E2) and D3-E2 group.

In the normal group, ACE was not detected in the cerebral cortex and medulla, diencephalon, hippocampus, or corpus striatum. In the vehicle group, ACE was not detected in the cerebral cortex and medulla or corpus striatum. In the D3-E2 and D7-E2 groups, the ACE concentration in each brain region tended to be higher than that in the corresponding regions of the normal and vehicle mice and was significantly (*p* < 0.05) higher in the midbrain (*) (Table 1). We measured ACE from the whole brain of experimental animals immediately after they were delivered from the supplier and found ACE in 3/3 mice tested (0.003394625 nmol/mg, 0.00103855 nmol/mg, and 0.001062725 nmol/mg).

**Figure 3:**
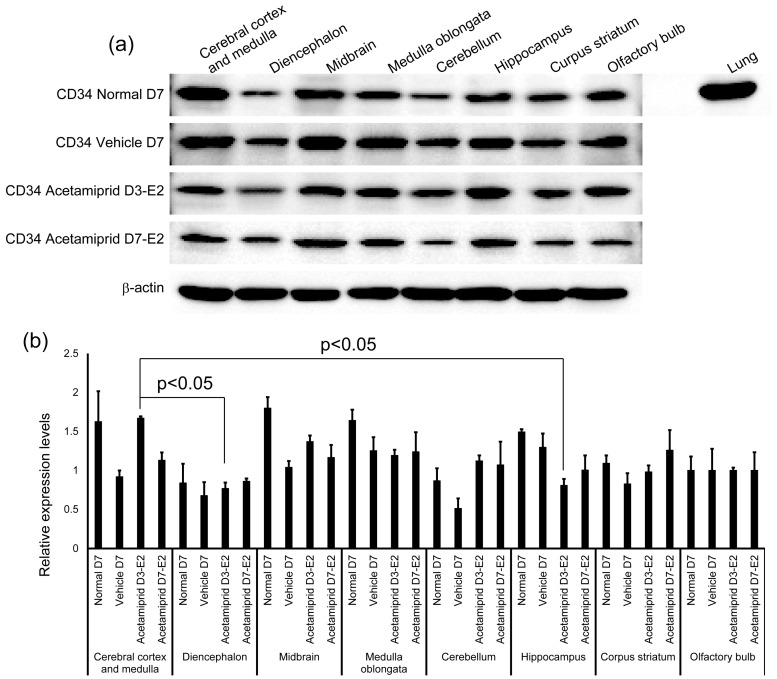
Expression of CD34 in different brain regions of normal, vehicle, and acetamiprid-treated mice. Western blot results are shown in (**a**); the lung is used as a positive control for the CD34 antibody. Relative intensity was calculated by which the expression of CD34 in the olfactory bulb for each point was normalized to 1; (**b**) each bar represents the mean ± SE. Values denoted as *p* < 0.05 were considered to be significantly different from the normal D7.

The expression of CD34 in normal, vehicle, and ACE-treated mice (D3-E2 and D7-E2) was calculated in terms of relative intensity, where the expression of CD34 in the normal mice for each point was normalized to one. Expression of CD34 in the D3-E2 group was significantly lower in the diencephalon and hippocampus than in the cerebral cortex and medulla (Figure 3).

**Figure 4:**
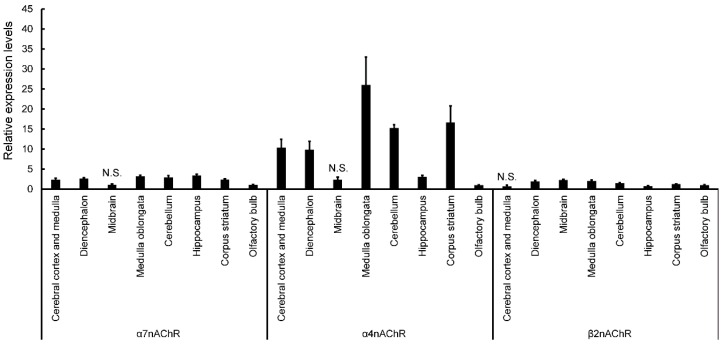
Expression of nicotinic acetylcholine receptors in different brain regions of normal mice. Relative intensity was calculated by which the expression of nAChRs in the olfactory bulb for each point was normalized to 1. Each bar represents the mean ± SE. Unless denoted as not significant (N.S.), the values are significantly different from the vehicle olfactory bulb (*p* < 0.05).

**Figure 5:**
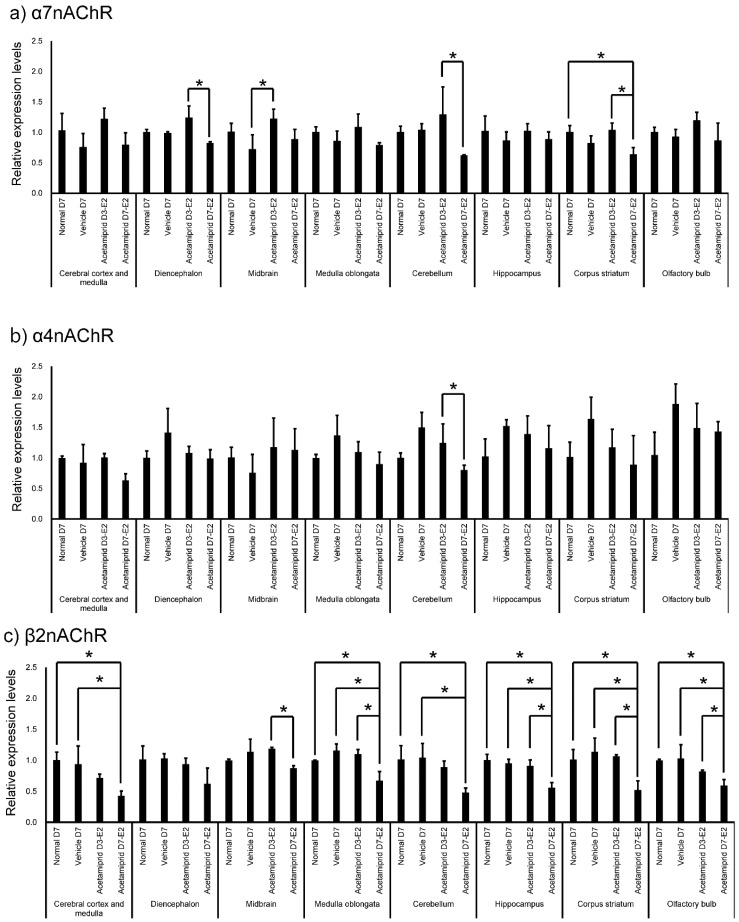
Expression of nicotinic acetylcholine receptors in different brain regions of normal, vehicle, and acetamiprid-treated mice. Relative intensity was calculated by which the expression of α7 (**a**), α4 (**b**), β2 (**c**) nAChRs for each point was normalized to 1. Each bar represents the mean ± SE. Asterisks (*) represent a significant difference, *p* < 0.05.

Expression of nAChRs in normal mice was calculated in terms of relative intensity, where the expression of nAChRs in the olfactory bulb for each point was normalized to one (Figure 4). Expression of α7nAChR was significantly higher in the cerebral cortex and medulla, diencephalon, medulla oblongata, cerebellum, hippocampus, and corpus striatum (except midbrain) than in the olfactory bulb (Figure 4). Similarly, expression of α4nAChR was significantly higher in the cerebral cortex and medulla, diencephalon, medulla oblongata, cerebellum, hippocampus, and corpus striatum (except midbrain) than in the olfactory bulb (Figure 4). Expression of β2nAChRs was significantly higher in the diencephalon, midbrain, medulla oblongata, cerebellum, and corpus striatum (except cerebral cortex and medulla) than in the olfactory bulb (Figure 4). Finally, expression of β2nAChRs was significantly lower in the hippocampus than in the olfactory bulb (Figure 4). The expression of nAChRs in normal, vehicle, and ACE-treated mice was calculated in terms of relative intensity by which the expression of nAChRs in the normal mice for each brain region was normalized to one (Figure 5). Expression of α7nAChR in the diencephalon and cerebellum was significantly lower in the D7-E2 group than in the D3-E2. Furthermore, expression of α7nAChR in the corpus striatum was significantly lower in the D7-E2 group than the normal D7 and D3-E2 groups (Figure 5). Expression of α7nAChR in the cerebellum was significantly higher in the D3-E2 group than in the vehicle group (Figure 5). Expression of α4nAChR in the cerebellum was significantly lower in the D7-E2 group than in the D3-E2 (Figure 5). Expression of β2nAChRs in the cerebral cortex and medulla, medulla oblongata, cerebellum, hippocampus, corpus striatum, and the olfactory bulb was significantly lower in the D7-E2 group than in the normal and vehicle groups (Figure 5). Expression of β2nAChRs in the midbrain, medulla oblongata, hippocampus, corpus striatum, and the olfactory bulb was significantly lower in the D7-E2 group than in the D3-E2 group (Figure 5).

## 4. Discussion

Exposure to ACE-containing water for 3 or 7 days (at decuple and centuple of NOAEL/day) caused a decrease in the body weight of mice but did not affect brain histology. In addition, ACE treatment did not affect expression of CD34. As mentioned earlier, the ACE concentration in each brain region of ACE-exposed mice tended to be higher than in the corresponding regions of the normal and vehicle mice. In particular, ACE concentrations were significantly higher in the midbrain of ACE-treated mice. However, expression levels of α7, α4, and β2 nAChRs were found to be low in the midbrain and the olfactory bulb of normal mice. Furthermore, expression of β2 nAChRs in the D7-E2 group decreased in many brain regions.

This study is the first to demonstrate nAChR expression and accumulation of neonicotinoid pesticides in mammalian brain regions. The concentration of ACE used in the present study, however, was much higher than that expected from exposure in a natural environment. It is possible that the ACE-containing diet used in this study exerted acute toxic effects on the liver and intestinal tract, resulting in hepatomegaly and diarrhea. Weight loss could have been attributable to diarrhea in ACE-treated mice.

Kapoor et al. [20] indicated that exposure to high doses of imidacloprid (20 mg/kg/day) for 90 days produced significant decreases in superoxide dismutase (SOD), catalase (CAT), and glutathione peroxidase (GPx) activities in the brains of female rats. Bhardwaj et al. [21] also reported that repeated exposure to high doses of imidacloprid (20 mg/kg/day) for 90 days produced necrotic Purkinje cells with loss of dendrites and granules in the granular layer of the cerebellum in the brains of female rats. This suggests a mechanism by which chronic exposure to neonicotinoids affects the brain. In this study, the exposure period to ACE was short and morphological changes were not observed.

However, offspring of rats exposed to a single intraperitoneal injection of imidacloprid (337 mg/kg/day) at day 9 of pregnancy exhibited significant sensorimotor impairments during behavioral assessments (at postnatal day 3). These impairments were associated with increased acetyl cholinesterase (AChE) activity in the midbrain, cortex, and brainstem (125%–145% increase), as well as the plasma (125% increase). However, histopathological evaluation using creosyl violet staining did not show any alteration in surviving neurons in a number of brain regions (the motor cortex, septal hippocampus, and cerebellum) [22]. Thus, even though there may be no morphological changes associated with a given level of ACE exposure, behavioral changes and sensory impairments may still be induced.

In one in vivo experiment, ACE, imidacloprid, and thiacloprid were separately administered by intraperitoneal injection to mice at 10 mg/kg [1,2]. Levels of the neonicotinoids in the mice brains were then measured at different time-points. Imidacloprid levels were 6.2 ppm at 15 min, declining to approximately 3 ppm at 60 min, and 1.5 ppm at 240 min. The level of thiacloprid at 15 min was 11 ppm, declining to 3 ppm at 60 min, and 0.8 ppm at 240 min. However, the amount of ACE increased from 1.3 ppm at 15 min to 3.3 ppm at 240 min. In other words, ACE was metabolized slowly relative to the other neonicotinoids. This resulted in relatively high levels in the brain for up to 240 min after treatment. Persistence in other tissues was also greater for ACE than for the other neonicotinoids [1,2]. Therefore, chronic exposure to ACE, more so than other neonicotinoids, may negatively affect a number of organs. This study [1,2] further confirmed that ACE, imidacloprid, and thiacloprid can pass through the blood–brain barrier. We hypothesize that ACE readily accumulates in the brain, and this is supported by the results of our study.

A recent report [18] described the effects of ACE exposure in patients from a clinical perspective. Exposure occurred during a period in which ACE (0.02%) was aerially sprayed over a pine forest in Japan approximately 55 times in a single month. A total of 78 patients from the nearby neighborhood complained of unexplained adverse effects to their physical condition. Details of reported symptoms were as follows: CNS symptoms (91%: cephalalgia, general malaise, depression, poor concentration, sleep disorders, memory impairment, irritability, and language disorders), skeletal muscle symptoms (91%: shoulder stiffness, muscle pain, muscle atrophy, muscle weakness, and tremors), and abnormal electrocardiograms (89%: ST–T changes, bradycardia, tachycardia, T wave abnormalities, and QT prolongation) [18]. Todani et al. [15] also reported that acute ACE poisoning in Japan can be accompanied by CNS symptoms, tachycardia, and muscle weakness. Imamura et al. [16] reported two cases of acute poisoning when an insecticide formulation containing ACE was ingested during suicide attempts. Symptoms that were common to these two cases included severe nausea, vomiting, muscle weakness, hypothermia, convulsions, tachycardia, and abnormal electrocardiograms [16]. Therefore, to the best of our knowledge, we conclude that symptoms that are common to ACE exposure include CNS symptoms, skeletal muscle disorders, and abnormal electrocardiograms.

In this study, expression of α4 and 7nAChR was significantly higher in the cerebral cortex and medulla, diencephalon, medulla oblongata, cerebellum, hippocampus, and corpus striatum (except midbrain) than in the olfactory bulb. Expression of β2nAChRs was significantly higher in the diencephalon, midbrain, medulla oblongata, cerebellum, and corpus striatum (except cerebral cortex and medulla) than in the olfactory bulb. The β2 and α7 subunits have been reported to have significantly higher expression in the hippocampus than other nAChR, and our data did not differ significantly [23]. Since there were no significant differences in the midbrain and olfactory bulb expression of α4 and 7nAChR, the olfactory bulb and midbrain may be less susceptible to potential adverse effects associated with ACE. In the D7-E2 group, expression of nAChRs decreased in many brain regions, particularly the β2nAChR. It has been shown that prolonged exposure to nicotine leads to an increase in the expression (upregulation) of nAChRs [24]. However, experiments comparing nicotine exposure by intravenous self-administration (SA) and passive subcutaneous nicotine administration via an osmotic minipump (MP) showed that α4β2 nAChRs were upregulated in all brain regions in the MP group, whereas α6β2 nAChRs were downregulated in the nucleus accumbens (NAc) and caudate-putamen [24]. Furthermore, α7 nAChRs were upregulated in the caudal cerebral cortex (CCx), and it was suggested that the α4β2α5 nAChRs were also upregulated in the CCx. In contrast, α4β2 upregulation was lower and limited to the CCx and NAc in the SA group; there were no detectable changes in α6β2 or α7 nACRs. Thus, expression levels of nAChRs appear to differ depending on the method of administration and brain region. In particular, the β2 receptor might be affected by ACE administration.

In the vehicle and normal mice, ACE was not detectable in the cerebral cortex and medulla, diencephalon, hippocampus, or corpus striatum; these mice were bred and managed in a specific pathogen free (SPF) area. Currently, neonicotinoid pesticides, including ACE, are not only used on crops, but are also used in building materials and household insecticides. Moreover, neonicotinoid pesticides have been detected in the river [25,26]. Therefore, there is a possibility that the neonicotinoid pesticide exposures occur from a mixture of animal bedding, drinking water, and food. In fact, neonicotinoid pesticides have been detected in many foods and building materials in our living area [27]. It is also required to carry out under our living environment. ACE is ubiquitously present in the environment or in the diet, meaning that all animals are chronically exposed to an amount of drug that may affect expression of nACRs. Therefore, in this study, a small amount of exposure to ACE might have affected the observed change in the nACRs. In the future, we will examine the expression of nACRs in animals housed under conditions that are confirmed to be ACE-free.

ACE exposure did not affect expression of CD34, a marker of vascular endothelial cells. In normal mice, the expression of CD34 was not significantly changed in each brain region. There may be no relationship between ACE accumulation and the expression of CD34 in the brain. The blood–brain barrier (BBB) is a diffusion barrier, and it impedes the influx of most compounds from the blood to the brain. There are three key cellular elements of the brain microvasculature. These include BBB-endothelial cells, astrocyte end-feet, and pericytes [28,29]. Therefore, differences in levels of ACE accumulation in different brain regions of the vehicle and normal mice might be affected by structural differences in the BBB other than expression of CD34 [30].

In this study, the ACE concentration was significantly higher in the midbrain of the ACE-treated mice than the normal and vehicle groups. There have been reports of eye pupil and dizziness symptoms in some ACE patients [18], but there are few reports of symptoms associated with characteristic midbrain failure. Furthermore, expression of β2 nAChRs in the D7-E2 group decreased in many brain regions. Expression of α4 and 7nAChR was also lower in the midbrain. Thus, any impact of ACE on the midbrain may be influenced by a change in expression of the nAChR receptors.

## 5. Conclusions

Exposure to ACE can result in differential accumulation in different regions of the brain. Symptoms that are common to ACE exposure include CNS symptoms, skeletal muscle disorders, and abnormal electrocardiograms. However, these are not the typical characteristic symptoms associated with the region of brain where ACE has been shown to accumulate. One potential reason for this finding may be that the expression levels of nAChR are different in each different brain region. Furthermore, in the experimental group (centuple ACE-containing water for seven days), expression of the acetylcholine receptor had decreased in many brain regions. Thus, knowing both the concentration of accumulated ACE and the expression of the nAChR in each region of the brain is important for understanding the observed clinical symptoms.

## Figures and Tables

**Table 1:** Concentration of acetamiprid in various brain regions in normal, vehicle, and acetamiprid-treated mice. In the D3-E2 and D7-E2 groups, the ACE concentration in each brain region tended to be higher than that in the corresponding regions of the normal and vehicle mice and was significantly (*p* < 0.05) higher in the midbrain (*). N.D. represent a not detection.

Brain Region	Group	Average Concentration (nM/mg)	SE
Cerebral cortex and medulla	Normal	N.D.	N.D.
	Vehicle	N.D.	N.D.
	Acetamiprid D3-E2	0.3954	0.3809
	Acetamiprid D7-E2	1.7158	2.2935
Diencephalon	Normal	N.D.	N.D.
	Vehicle	0.0087	0.0124
	Acetamiprid D3-E2	1.9417	3.6850
	Acetamiprid D7-E2	0.5414	0.7269
Midbrain	Normal	0.1825	0.2807
	Vehicle	0.4916	0.0813
	Acetamiprid D3-E2	1.1390 *	0.4940
	Acetamiprid D7-E2	1.3837 *	0.8010
Medulla oblongata	Normal	1.9539	0.5117
	Vehicle	1.6952	0.8775
	Acetamiprid D3-E2	2.0929	2.2027
	Acetamiprid D7-E2	1.6584	1.0396
Cerebellum	Normal	0.1015	0.1404
	Vehicle	0.1935	0.0434
	Acetamiprid D3-E2	1.0206	0.7893
	Acetamiprid D7-E2	0.7685	0.7817
Hippocampus	Normal	N.D.	N.D.
	Vehicle	0.2642	0.3736
	Acetamiprid D3-E2	1.0159	2.2716
	Acetamiprid D7-E2	1.9809	2.6540
Corpus striatum	Normal	N.D.	N.D.
	Vehicle	N.D.	N.D.
	Acetamiprid D3-E2	1.4060	1.1609
	Acetamiprid D7-E2	3.3641	4.5826
Olfactory bulb	Normal	3.1730	1.5092
	Vehicle	4.4309	0.2696
	Acetamiprid D3-E2	7.3065	5.8516
	Acetamiprid D7-E2	5.3325	2.0955

## Supplementary Materials

The following are available online at www.mdpi.com/1660-4601/13/10/937/s1, Table S1: Real time RT-PCR primer’s list.
