# Supplementary material for: Acetamiprid Accumulates in Different Amounts in Murine Brain Regions
Source: Int J Environ Res Public Health. 2016 Sep 22;13(10):937. doi: 10.3390/ijerph13100937 (PMC5086676; doi:10.3390/ijerph13100937)
Supplement: Supplementary file 1 [file ijerph-13-00937-s001.pdf]

Supplementary Materials: Acetamiprid Accumulates in Different Amounts in Murine Brain Regions

Hayato Terayama, Hitoshi Endo, Hideo Tsukamoto, Koichi Matsumoto, Mai Umezu, Teruhisa Kanazawa, Masatoshi Ito, Tadayuki Sato, Munekazu Naito, Satoshi Kawakami, Yasuhiro Fujino, Masayuki Tatemichi and Kou Sakabe

Table S1. Real-time RT-PCR primer list.

Primer Name	Direction	Sequence
α7nAChR	Forward	ctctgactgtcttcatgctgct
	Reverse	atcatggtgctggcgaag
α4nAChR	Forward	cgtccagtacattgcagacc
	Reverse	atgaccatggccacgtattt
β2nAChR	Forward	actctatggcgctgctgttc
	Reverse	tcctctgtgtcagtacccaaaa

© 2016 by the authors; licensee MDPI, Basel, Switzerland. This article is an open access article distributed under the terms and conditions of the Creative Commons by Attribution (CC-BY) license (http://creativecommons.org/licenses/by/4.0/).
